# Influence of Embedded Gap and Overlap Fiber Placement Defects on Interlaminar Properties of High Performance Composites

**DOI:** 10.3390/ma14185332

**Published:** 2021-09-15

**Authors:** Denis Cartié, Marine Lan, Peter Davies, Christophe Baley

**Affiliations:** 1 Coriolis Composites SAS, Rue Condorcet, Z.A. du Mourillon, 56350 Quéven, France; 2 IRDL, Université de Bretagne Sud, UMR CNRS 6027, Rue de Saint Maudé, 56000 Lorient, France; lan.marine.lan@gmail.com (M.L.); christophe.baley@univ-ubs.fr (C.B.); 3 Ifremer, Centre Bretagne, Technopôle Brest Iroise, 29280 Plouzané, France; peter.davies@ifremer.fr

**Keywords:** fiber placement, gap, overlap, interlaminar, delamination

## Abstract

Automated fiber placement (AFP), once limited to aerospace, is gaining acceptance and offers great potential for marine structures. This paper describes the influence of manufacturing defects, gaps, and overlaps, on the out-of-plane properties of carbon/epoxy composites manufactured by AFP. Apparent interlaminar shear strength measured by short beam shear tests was not affected by the presence of defects. However, the defects do affect delamination propagation. Under Mode I (tension) loading a small crack arrest effect is noted, resulting in higher apparent fracture energies, particularly for specimens manufactured using a caul plate. Under Mode II (in-plane shear) loading there is a more significant effect with increased fracture resistance, as stable propagation for specimens with small gaps changes to arrest with unstable propagation for larger gaps.

## 1. Introduction

**Figure 1:**
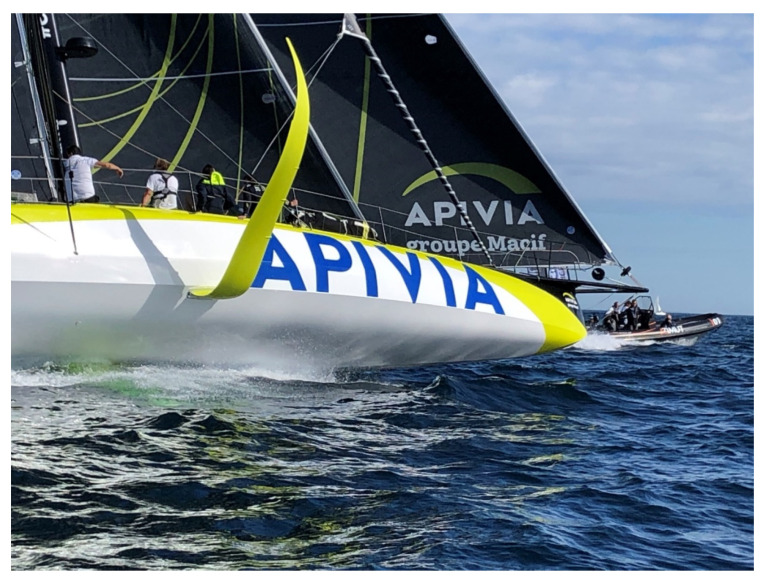
Photograph of IMOCA class APIVIA racing boat, the hydrofoils are manufactured using AFP technology: Photo C. Baley.

The use of automated fiber placement (AFP) is increasing, as it offers the possibility to produce very complex shapes with tight process control [1]. Initially developed for high performance aerospace applications, the capability for efficient manufacture of complex structures can also be applied to marine components such as hydrofoils [2], propellers [3], and tidal turbine blades [4]. These structures tend to be thicker than aerospace composites, so through-thickness properties are more critical. Figure 1 shows an example of a foil manufactured by AFP on an ocean racing yacht.

AFP enables the trajectory of unidirectional composite tape to be optimized but laying down complex shapes with this technology can result in defect introduction. Two particular types of defects are possible; gaps between tapes and overlaps where they are superposed. Several authors have investigated the influence of these defects on in-plane properties, with particular emphasis on the more critical compression properties. These have included studies to quantify how 90° defects can result in ply waviness, leading to reduced compression performance [5], and tow drops have also been shown to affect compression behaviour [6]. The number and offset of gaps are additional parameters which have been examined [7,8,9,10]. Experimental studies have also underlined the importance of defect orientation [11,12]. A recent study highlighted the importance of staggering, offsetting successive ply defects, to reduce their influence [13]. There has also been some testing and modelling work on the influence of gaps on behaviour under dynamic loading [14,15] and fatigue [16]. In two previous papers, for the same carbon-epoxy material as studied here, the authors also examined the influence of different gap and overlap singularities first under in-plane tensile [17], then under in-plane shear and compression loads [18]. Such defects may occur due to variations in tape width or movements on complex mould shapes. Samples were produced with and without caul plates. Under certain conditions, the caul plate can promote healing of defects. For example, after autoclave cure of blocks of plies with one orientation no defects remain when a caul plate is present, only a local change in thickness. There is then no loss in tensile properties. In blocks of plies with different orientations, the effect of the caul plate is less significant, but changes in thickness are still reduced. Under compression loads, when buckling is present, the presence of a caul plate during cure helps to retain properties.

In spite of the significant amount of data now available for in-plane properties very few studies have focused on the influence of manufacturing defects on out-of-plane behaviour. Comer and colleagues [19,20] did study the interlaminar fracture behaviour of thermoplastic composites manufactured by tape placement but without defects. They found that the laminates produced by Laser Assisted Automated Tape Placement (LATP) performed better than the autoclaved laminates in terms of interlaminar fracture toughness, probably due to the presence of butt joints, but ILSS (interlaminar shear strength) and other mechanical properties were lower. Void contents were higher in the LATP materials. Stokes-Griffin and Compston [21] also used an out-of-plane shear test (ILSS) to study processing parameters for tape placement of carbon/PEEK and found a significant influence of placement rate. Grouve et al. [22] used a peel test to examine adhesion between layers in tape laid carbon/PPS laminates for different manufacturing conditions, but again without studying singularities. Other work on the influence of manufacturing singularities on out of plane properties include a recent study by Zhou et al. who used a through thickness tensile test [23]. Their experimental and numerical results indicated that gaps of up to 3 mm could result in a drop in out-of-plane tensile strength from around 37 to 30 MPa. Ghayour et al. used short and long flexural specimens to examine the effect of tow gaps. They found a reduction in apparent shear strength of 13% and a drop in flexural stiffness of 35% (due to thickness reduction), compared with a hand lay-up reference, when gaps were present [24]. However, to date there has been very little work to characterize the presence of defects in AFP composites using a fracture mechanics approach. This is a powerful method to enable the interaction of local singularities with a propagating crack to be characterized, and fracture mechanics values are being increasingly integrated in design in order to evaluate damage tolerance. A recent paper describes these tests [25].

This paper will examine the influence of singularities deliberately introduced at the mid plane of [90/0_7_/90]_S_ laminates on the out-of-plane fracture properties of the same carbon/epoxy composites as those previously studied under in-plane loading [17,18]. Samples from plates manufactured with and without caul plates were tested. This is an original application of fracture mechanics testing, which introduces some difficulties in specimen definition and analysis but provides the basis to allow the presence of defects to be accounted for in design.

## 2. Material and Methods

### 2.1. Material

The results reported in this paper were obtained by testing laminates made from AS4/8552 prepreg supplied by Hexcel Composites in Dagneux (France), reference (8552/AS4/RC34/AW194). Various plates were manufactured using a Coriolis 8 tow robotic fibre placement machine installed at Quéven (France) [26]. In order to be processable by the machine, the prepreg is slit to a width of 6.35 ± 0.125 mm. A single batch of prepreg material was employed to manufacture all the panels, with a compaction force of 600 N applied during lay-up. In order to introduce gap and overlap defects in specific regions of the panels, the machine was programmed using off-line software to stagger sectors. The same defect sizes as references [17,18] were introduced as they can be commonly found in double curvature parts produced using the AFP technology.

A 15 μm thick PTFE film was inserted at the mid-plane of the laminate to act as a delamination initiator. The edge of the starter film was located 10 mm from the centre of the gap/overlap defects.

In order to determine if the caul plate has an effect on the delamination behaviour of laminates having manufacturing singularities, a first series of panels was cured using a caul plate (2 mm thick aluminium sheet) while a second series of panels was cured without. To promote flow of material during cure and prevent the laminate from sticking to the caul plate, a Wrightlon™ 5200 PTFE release film supplied by AirTech^®^ (Springfield, TN, USA) was placed between the plate and the laminate.

The panels were cured in an autoclave at 180 °C under 7 bar pressure for 2 h after a dwell at 110 °C for 1 h, following the prepreg supplier’s recommendations. After cure, the panels were C-scanned to check the quality of the laminates. The test specimens were then cut using a diamond coated wet circular saw and the edges were polished to prevent premature crack initiation.

### 2.2. Configuration of Samples

Two types of tests were employed in this study. First, short beam shear tests were performed on specimens from panels manufactured with a caul plate. This is a widely-used quality control test described by ASTM D2344. Then Mode I and Mode II interlaminar fracture tests were performed. These are usually performed on unidirectional laminates, but in order to investigate how crack propagation is affected by gap or overlap defects, these singularities must be placed in a layer at 90° to the crack front. This requires careful consideration; Mode III stresses may occur during the loading phase if both the specimen and each specimen arm are not symmetrical with respect to their mid-planes. Anticlastic deformation will be generated by the non-symmetrical half laminates. 

This has been addressed in previous studies on delamination of cross-ply laminates [27,28,29]. The solution adopted here was to balance each arm by placing an additional 90° layer on the outer surface of the specimen, so the specimen layup was the following:

[90°/0°_7_/90°_2_/0°_7_/90°]

The samples must also be stiff enough to avoid large displacements, and this was achieved by keeping the majority of unidirectional plies in each arm of the laminate.

**Figure 2:**
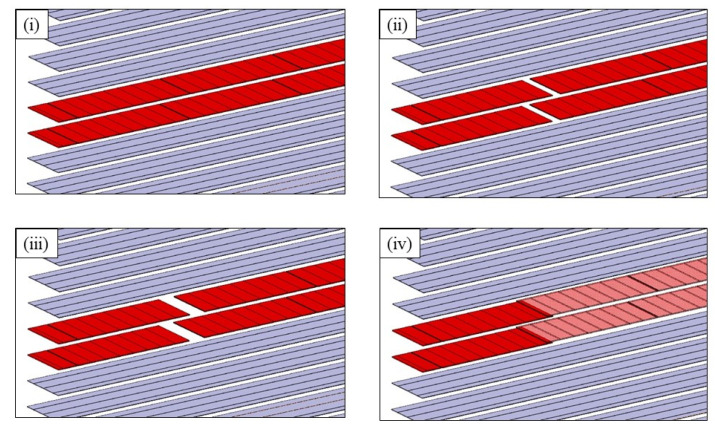
Specimens layups [90°/0°7/90°2/0°7/90°] showing embedded defects in the centre plies of the laminate. Plies oriented at 0° are shown in grey and plies oriented at 90° are shown in red. (**i**) Gap 0.5 mm; (**ii**) Gap 3.175 mm, (**iii**) Gap 6.35 mm, (**iv**) Overlap 3.175 mm.

Figure 2 shows schematically the defect positions in the two central 90° plies.

### 2.3. Material Quality Control

All test panels were inspected using ultrasonic C-Scan. Sofratest™ 49,944 equipment was used for the inspections with a flat aluminium panel acting as a reflector. The control was performed by a focused transducer, with a frequency of 10 MHz and a focalization length of 76 mm. The acquisition step was 0.5 mm. The quality of all the panels was satisfactory, with low attenuation and no evidence of delamination.

**Figure 3:**
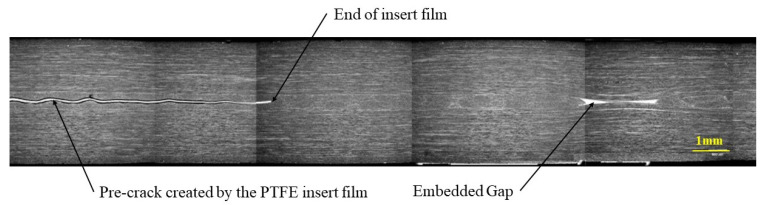
Microstructural SEM observation of a specimen containing embedded defect used for delamination tests—Gap 6.35 mm with caul plate.

To check for voids and to verify the position and the morphology of the embedded defects, cross sections were observed with a Jeol JSM 6460 LV scanning electron microscope. To produce flat sections, the samples were polished with diamond paste down to 1 micron before examination. Several images were taken and then assembled, to produce the figures of defect regions shown in this paper. Figure 3 shows a section through the thickness of a specimen, illustrating the film insert and a 6.35 mm gap. The pre-crack created by the PTFE film is clearly visible on the left-hand side of the micro-graph. It can be noted that the defect initially created by a 6.35 mm gap has healed during cure. The resin and the 90° fibres have flowed into the gap reducing the defect size to about 2 mm.

### 2.4. Short Beam Shear Tests

Short beam shear tests (NF EN ISO 14130) were performed in three-point flexure under displacement control at 2 mm/minute on specimens from all the panels produced with a caul plate. The distance between supports was 5 times the specimen thickness. Apparent interlaminar shear strength τ_13_ is calculated as: 0.75*P/(Bh), with P the critical load at delamination, B the specimen width, and h the thickness.

### 2.5. Interlaminar Fracture Toughness Tests

The delamination specimens were tested on an Instron™ test machine with a load cell of 10 kN at loading rates of 2 mm/min and 1 mm/min respectively for Mode I and Mode II tests. For each configuration, an average of six specimens was tested. 

The configuration of the delamination tests in Mode I is the Double Cantilever Beam which respects the standard ISO15024 [30]. This test applies a through-the-thickness tension to the two arms of samples. The loading is introduced through aluminium blocks bonded to the end of the samples on the upper and lower surfaces. Specimens are loaded by pins that leave the blocks free to rotate. Cyanoacrylate adhesive was used to bond the blocks.

**Figure 4:**
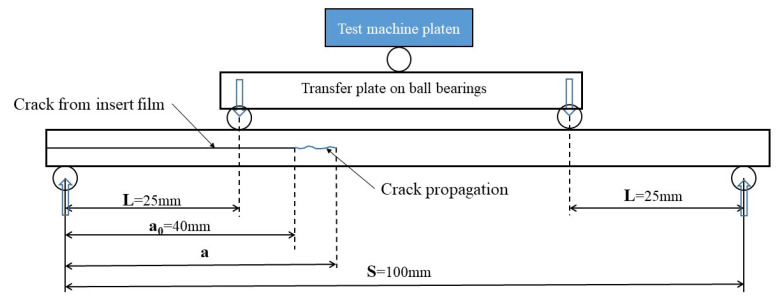
Mode II 4-ENF specimen configuration.

For Mode II delamination tests a 4ENF (Four Point End Notched Flexure) geometry [31] was used to propagate mid-plane cracks under the effect of a shear stress introduced in 4-point bending. This is one of a number of in-plane shear delamination tests available [32] and has the advantage of encouraging the stable crack propagation required here. Figure 4 illustrates the 4ENF specimen configuration. For this test, the distance between the upper loading points was 50 mm and the distance between the lower supports was 100 mm. A roller bearing was positioned so that the upper load points rotate about the specimen mid-length.

### 2.6. Data Analysis

Standard data analysis to determine strain energy release rates was not applicable here because of the cross-ply nature of the sample and the unstable behaviour of the crack growth. The method used is based on the calculation of the crack length using beam theory and requires only the force and displacement data. This data analysis was developed by the author in a previous study of delamination tests performed under pressure [33] when visual crack length measurement was not possible inside a pressure vessel. The derivation of the data analysis can be found in [33]. 

For Mode I the crack length is calculated using the following equation:

(1) aCalc=[3EIδ2P]13

where *a_Calc_* is the calculated crack length, *E* is the flexural modulus, *I* is the second moment of inertia, *δ* is the opening displacement and *P* the applied load. 

The apparent fracture toughness in Mode I, G_Iapp_, is then calculated as:

(2) GIapp=1b[3P2δ2EI]23

For Mode II the crack length is calculated as:

(3) $$a_{Calc}=\frac{1}{3}\left[\frac{32EI\delta }{PL^{2}}+\frac{10}{3}L-S\right]$$

Here, *δ* is the deflection of the beam due to cross head displacement, *P* is the applied load, *E* is the flexural modulus, *I* is the second moment of inertia, *S* the span between the outer loading rollers, and *L* the distance between the inner and the outer loading rollers.

The apparent fracture toughness in Mode II is then calculated as:

(4) $$G_{\mathrm{IIapp}}=\frac{P^{2}}{2b}\frac{3L^{2}}{32EI}$$

It should be noted that the crack lengths and toughness values given in this paper are apparent values. They can only be used in a comparative way, but they enable the effect of the gap/overlap defects on the delamination behaviour of CF/epoxy laminates to be examined.

For calculations under both Mode I and Mode II loading, *δ* and *P* were recorded by the test machine data acquisition system.

In order to determine the term *EI*, Equations (1) and (3) were inverted giving the two following equations:

(5) $$EI=\frac{P}{\delta }\frac{2}{3}a_{0}^{3}=\frac{1}{C}\frac{2}{3}a_{0}^{3}$$

(6) $$EI=\frac{P}{\delta }\frac{L^{2}}{32}\left[3a_{0}+S-\frac{10}{3}L\right]=\frac{1}{C}\frac{L^{2}}{32}\left[3a_{0}+S-\frac{10}{3}L\right]$$

During the linear loading phase of the test prior to any crack propagation, the initial crack length *a*_0_ is known (See Figure 4). The compliance *C = δ/P* is determined from the slope of the force vs. displacement curve during the linear loading, allowing *EI* to be determined for each specimen. Then *G_IIcApp_* can be determined from Equation (4).

## 3. Results

### 3.1. Influence of Singularities on Interlaminar Shear Strength

**Figure 5:**
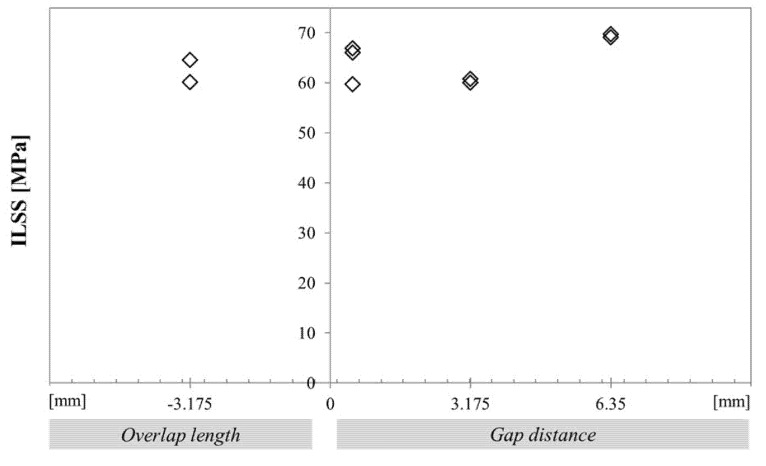
Apparent Interlaminar shear stress variation as a function of the type of embedded defect with caul plate, [90/0_7_/90_2_/0_7_/90].

Figure 5 shows the results from short beam shear tests. These results indicate that the short beam shear test, among the most widely used tests to check composite quality, is not very sensitive to the presence of these singularities. This is an interesting result, but this test has some limitations as noted by Whitney and Browning [34]. They showed the complex stress state in ILSS specimens, with compression stresses tended to suppress the interlaminar shear failure mode. It was therefore decided to perform interlaminar fracture tests, as these can quantify the behaviour when a propagating delamination meets a zone containing singularities.

### 3.2. Mode I Testing of Unidirectional Composites without Singularities

**Figure 6:**
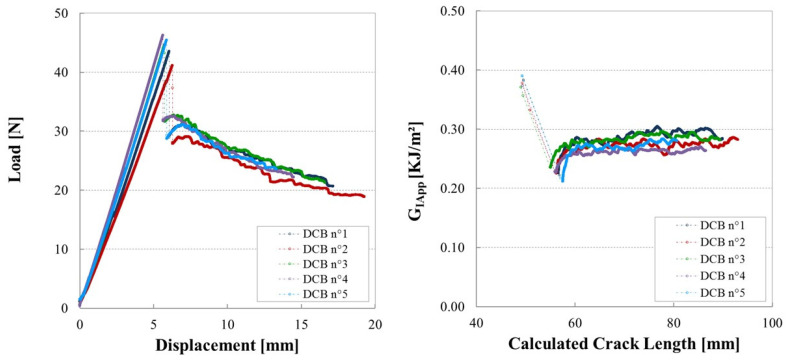
Mode I fracture test results for unidirectional samples without singularities. (**Left**) Force–displacement. (**Right**) Apparent G_Ic_ versus crack length.

Figure 6 shows the results from Mode I tests on samples laid up from prepreg layers of the same material as that employed for tape laying, and manufactured in the autoclave with the same cure cycle, i.e., the same composite but without any singularities.

These curves show typical behaviour of this material in DCB tests without singularities. The initiation from the starter film is unstable for all specimens, a load drop is noted on the force–displacement plot as the crack rapidly advances a few millimetres beyond the insert film, but then stable propagation is recorded throughout the test. This results in propagation values in the range 0.25–0.30 kJ/m^2^.

### 3.3. Influence of Manufacturing Defects in Mode I

**Figure 7:**
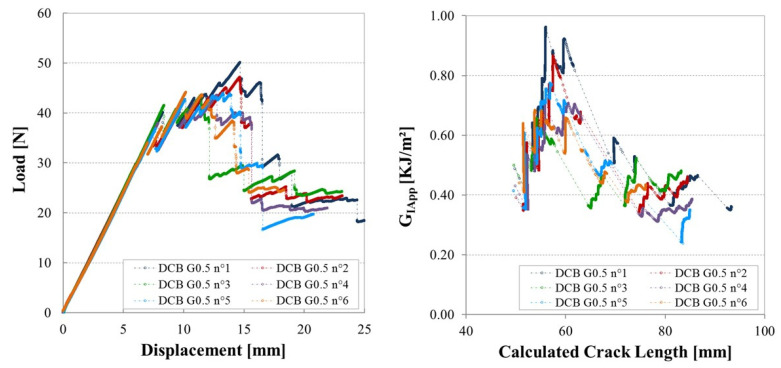
Load vs. displacement traces (**left**) and R-curves (**right**) for cross ply laminates ([90/0_7_/90]s) containing a 0.5 mm gap (considered as reference configuration without defects).

Figure 7 shows the load vs. displacement traces (left) and the calculated R-curves (right) of [90/0_7_/90]s laminates containing 0.5 mm gaps. The crack propagation is typical of stick/slip behaviour. The crack jumps immediately to a 90/0 interface. With further loading, the crack jumps from one 90/0 interface to the other 90/0 interface. This phenomenon has also been observed by Brunner and Blackman for tests on specimens with 90° central layers without singularities [35]. Despite the differences in the laminate configurations, the apparent toughness levels reached in this study are comparable with the calculated G_IC_ values reported by Brunner and Blackman.

**Figure 8:**
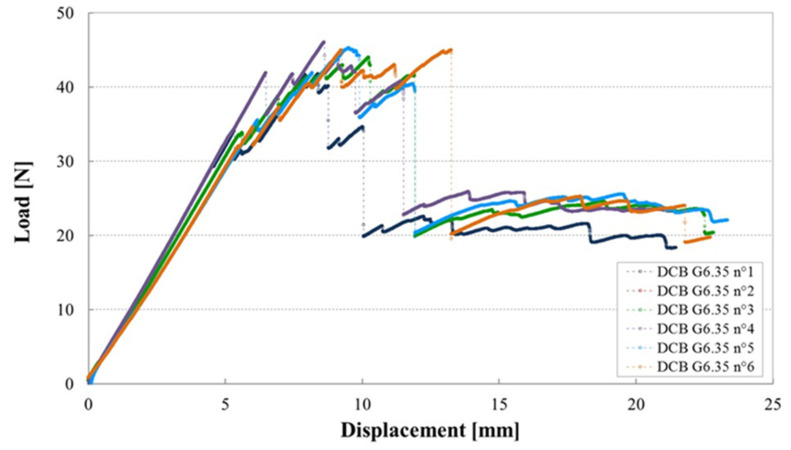
Load-displacement plots for the various samples containing a Gap of 6.35 mm polymerized with caul plate.

Figure 8 shows an example of the load/displacement plots from six DCB tests on specimens with the largest gap defects. There is an initial stable propagation as the damage zone in front of the insert develops, then the crack meets the singularity region and the load increases until an unstable crack jump. Further crack propagation is then stable. The plots from the six specimens all show the same form, with similar load levels once the crack has passed the singularity.

**Figure 9:**
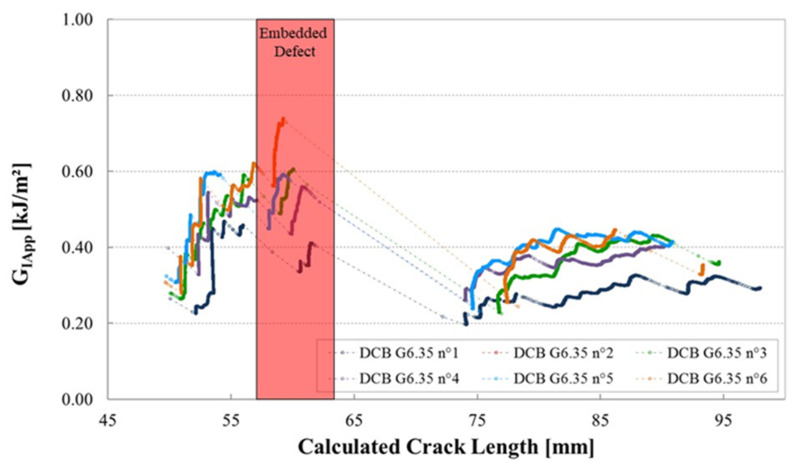
Mode I fracture R-curve, strain energy release rate versus the calculated crack length, for 6 DCB specimens containing a gap of 6.35 mm, manufactured with caul plate (calculated from plots in Figure 8).

The corresponding R-curves (Figure 9) show that G_IApp_ increases as the crack approaches the zone containing the manufacturing defect (red shaded area), then an unstable crack propagation of around 15 mm is noted, before the fracture energy stabilizes.

Table 1 summarizes the results from Mode I tests on specimens from panels with the eight singularity conditions. The values shown are mean values. For each type of defect, a peak value in the defect zone and a value at a calculated (arbitrary) crack length of 80 mm, i.e., beyond the singularity, are given.

### 3.4. Mode II Testing of Unidirectional Laminates without Singularities

**Figure 10:**
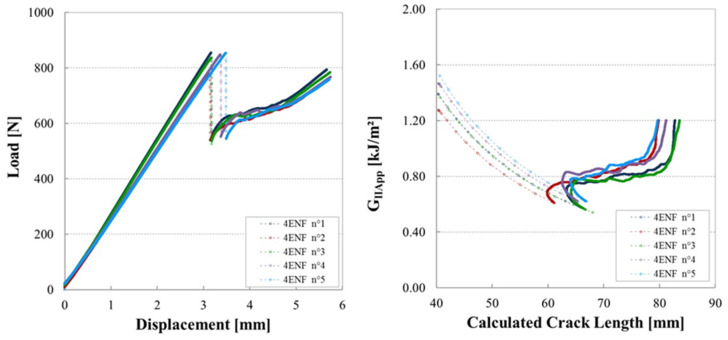
Load vs. displacement curves and R-curves of the Mode II unidirectional laminates.

Figure 10 shows the results from the Mode II tests on the same material in unidirectional form without singularities. As for the Mode I tests on unidirectional specimens (Figure 6), the initiation of the delamination is unstable. The subsequent crack propagation is stable reaching G_IIapp_ values of the order of 800 J/m^2^. This level of G_II__a__pp_ is comparable to results found in the literature for the same material (values around 0.9 kJ/m^2^) [36].

### 3.5. Influence of Manufacturing Defects in Mode II

**Figure 11:**
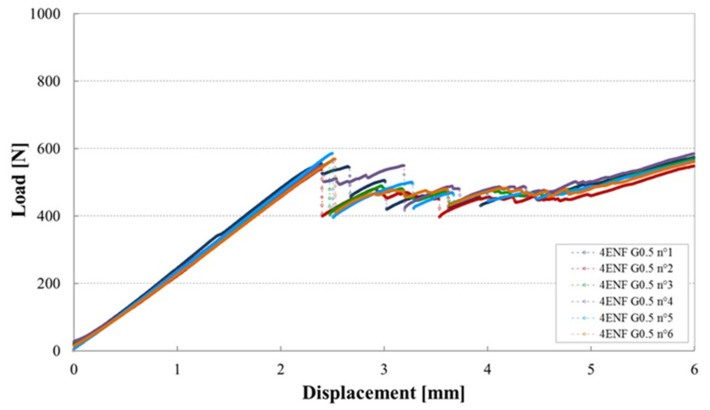
Load–displacement plots for tests on six 4ENF specimens containing a Gap of 0.5 mm manufactured with a caul plate.

Figure 11 shows examples of load–displacement plots for Mode II tests of the cross ply laminated containing a gap of 0.5 mm, in which no defects could be found after cure.

Here there is an initial load drop, followed by a period of stick–slip crack propagation, then a steep increase in load as the crack reached the region of the specimen affected by the loading point compression zone. The plots for the six specimens are quite similar, both in terms of behaviour and values.

**Figure 12:**
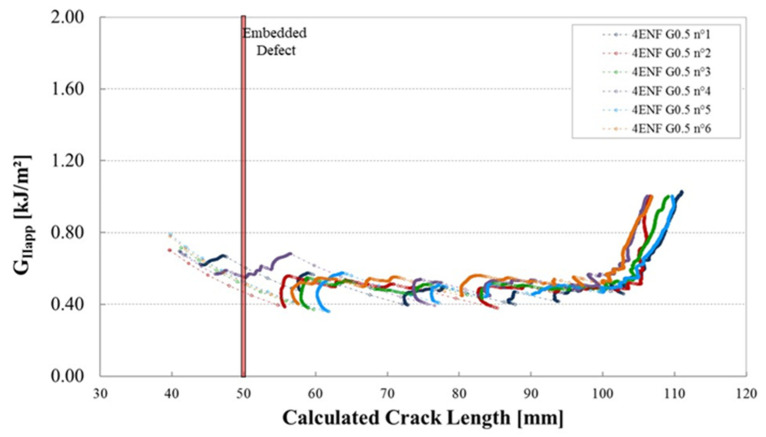
Mode II fracture energy as a function of the crack length for six 4ENF specimens containing a gap of 0.5 mm from a plate manufactured with caul plate (reference configuration considered without defects).

Figure 12 shows the corresponding plots of G_IIapp_ versus calculated crack length for these specimens. The shaded area represents the location and size of the embedded defect.

**Figure 13:**
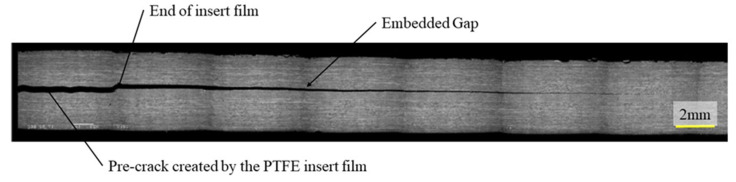
Example of a Mode II crack propagation path on a specimen with a 0.5 mm gap with caul plate.

**Figure 14:**
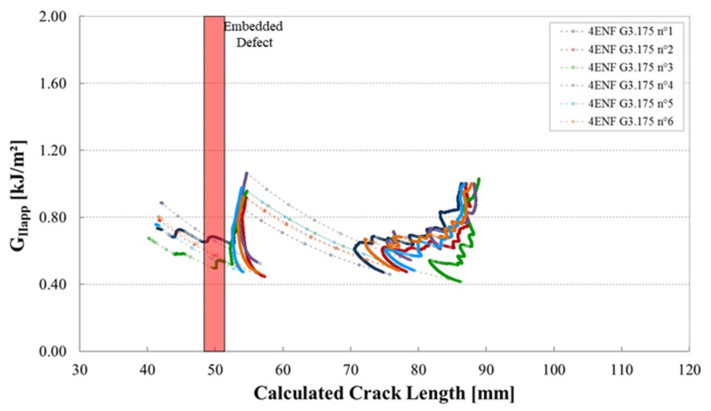
Mode II fracture energy as a function of the crack length for six 4ENF specimens containing a gap of 3.175 mm, plates manufactured with caul plate.

**Figure 15:**
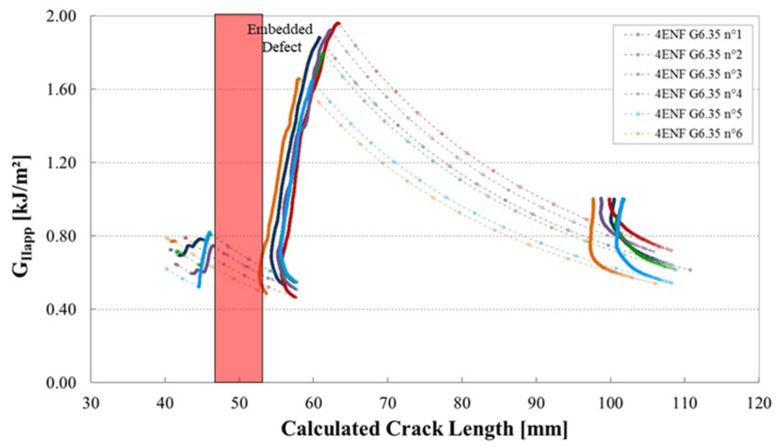
Mode II fracture energy as a function of the crack length for six 4 ENF specimens containing a gap of 6.35 mm from plates manufactured with caul plate.

**Figure 16:**
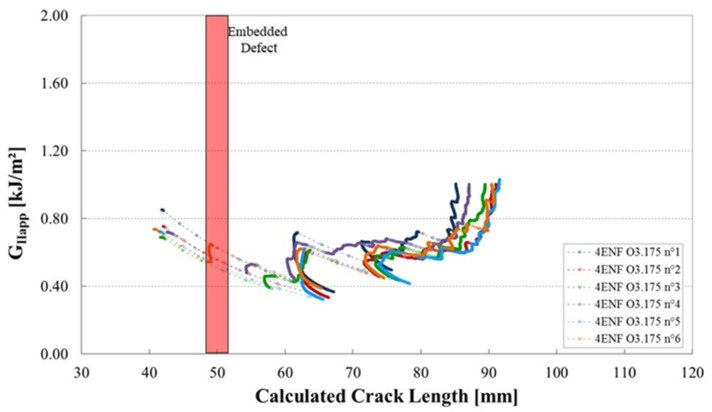
Mode II fracture energy as a function of the crack length for six 4ENF specimens containing an overlap of 3.175 mm from plates manufactured with caul plate.

The shape of the R-curves suggests that the stiffness of the sample increases after an unstable crack propagation as observed for the samples cut out of unidirectional laminates. The observation of the edges of the test coupons indicates that the crack front has jumped to the upper 0°/90° interface, as illustrated in Figure 13. In this case, corresponding to the smallest defect, there is an initial crack jump from the insert to the defect zone. Then from the defect zone onwards the propagation values appear to be quite stable, around 0.65 kJ/m^2^. Figure 14 and Figure 15 show the R-curves for six Mode II specimens with the two larger gaps (3.175 mm and 6.35 mm). A clear effect of the defect zone on the crack propagation behaviour is observed, with crack arrest, there is an increase in apparent fracture energy followed by an increasingly unstable jump as the defect zone is increased. For the 6.35 mm gap the crack jumps to below the loading point, there is no longer any stable propagation. The results for the 3.175 mm gap show an intermediate behaviour. Figure 16 shows the R-curves of a laminate containing a 3.175 mm overlap defect (Figure 2iv). The crack propagation in these samples differs from that of specimens containing gap type defects by the fact that the crack advances rapidly through the defect area to a calculated crack length of approximately 65 mm with a corresponding G_IIapp_ of approximately 0.4 kJ/m^2^. Once the crack front has passed the defect area, the crack propagates in a more stable manner at a G_IIapp_ of the order of 0.6 kJ/m^2^, similar to the values for specimens without defects, until it reaches the loading roller. The behaviour is similar to that of the specimens without defects but with more unstable crack propagation through the defect area. This can be explained by the fact that in the case of overlap defects, the local fibre content is increased. The lack of resin results in lower resistance to the crack propagation.

Table 2 summarizes the Mode II results. Once again, to compare the effects of the different defect types, the values are taken as the peak values and those corresponding to an arbitrary calculated crack length of 75 mm.

## 4. Discussion

First, it should be noted that this type of interlaminar fracture data for AFP composites with singularities does not exist in the literature, so it is not possible to compare results with published values. However, the tests provide a large amount of information which is discussed below in three sections: First the validity of the experimental approach is analysed. Then the influence of the type of loading on the crack propagation resistance is discussed. Fnally, the influence of the caul plate is examined.

The Mode I delamination test on unidirectional laminates is standardized, and has been extensively studied, but its application to AFP materials poses three main difficulties. The first is the need to balance the stacking sequence of the specimen arms, so that they are symmetric both with respect to the overall specimen and also with respect to each arm mid-plane. As the defects need to be placed in 90° layers, this requires adding an additional external 90° ply on each face resulting in a specific stacking sequence for the test. The second choice to be made is the position of the singularity as the aim here is to examine how a propagating crack is affected when it meets a singularity; this differs from the standard test which is primarily focused on obtaining initiation values of G_Ic_ from implanted thin films. Here a distance of 10 mm was maintained between the end of the insert film and the centre of the defect in order that the crack will start to propagate. This choice of distance is open to discussion; a longer distance may allow additional damage mechanisms to develop, while a shorter distance may be affected by the insert film. However, the distance was kept constant for all tests and should therefore allow direct comparisons between defect types to be made. Finally, the third difficulty is determining crack length. This is intrinsic to all fracture mechanics tests, but the choice here was to calculate the crack length from the measured force and displacement. Again, this is open to discussion but the same approach was used for all tests and so again it allows comparisons to be made for different defects on the same basis.

Mode II testing is more controversial than Mode I, even without adding manufacturing defects [32]. The choice here of a four-point ENF specimen was based on the need for a stable crack propagation for which this geometry is the simplest available option. Once again, the main difficulty is the determination of the crack length and the choice was made here to use the calculated apparent crack length value.

It is noticeable that in some cases the calculated crack length appears to decrease after the unstable crack propagation. This crack length is derived from the measured stiffness of the specimen, and the latter will depend on both the open crack and the damage zone in front of the crack. Aksoy and Carlsson [37] showed that this damage zone involves micro-cracks which extend well beyond the open crack and coalesce to form a new crack surface. The relationship between measured stiffness and crack length is further complicated by the friction between fracture surfaces, which has been shown to have a significant influence on Mode II fracture energy [38].

**Figure 17:**
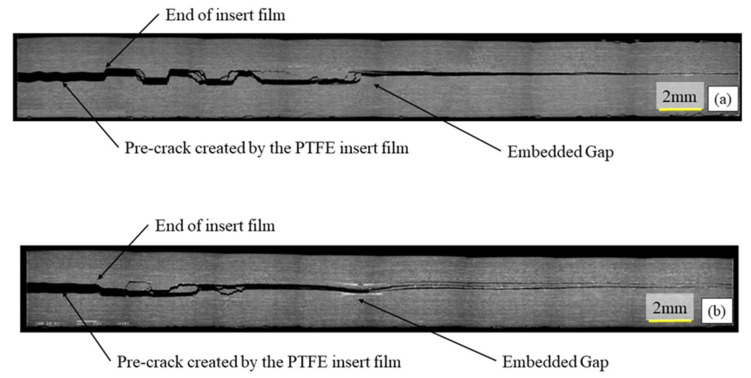
Examples of Mode I crack propagation paths for tests on (**a**) gap 0.5 mm, with caul plate, (**b**) gap 6.35 mm, with caul plate.

A comparison of the Mode I results with those for the unidirectional material indicates higher peak values in the initiation region. The apparent crack resistance tends to increase as the damage zone in front of the crack top interacts with the singularities. Various authors have shown that a plastic or process zone precedes the main crack tip during Mode I propagation [39,40]. As a result of the development of this zone, and its interaction with through-thickness defects, damage including resin cracking and debonding, can occur above and below the crack plane, initiating secondary cracks. Even during the first millimetres of propagation, there is a tendency for the crack to jump between the 0/90° interfaces (see Figure 17). This has been seen in previous work on crack propagation at 0/90° interfaces [41]. These appear to encourage crack bifurcation, deviating cracks to the 0/90° interface, and this raises the apparent fracture energy, Figure 17b. Once the crack has passed the singularity it propagates more easily, and stable propagation fracture energy values return to those measured at the start of the test, around 0.3–0.4 kJ/m^2^. These values are similar to those published elsewhere for this 0/90° material [41]. The type and dimensions of the singularity and the presence of a caul plate, all affect the peak fracture energy (Table 1).

**Figure 18:**
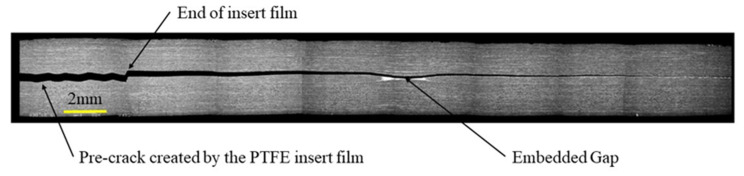
Micrograph Mode II specimen, 6.35 mm gap with caul plate.

Concerning the Mode II results, these clearly indicate that the presence of the larger gaps has a significant effect on the crack propagation behaviour. There is a large arresting effect increasing with increasing defect size (within the range of defect tested in this study) followed by unstable crack growth. The specimens with overlaps are much less affected by the defect. Examination of micrographs suggests that the crack arrest may be due to large resin pockets resisting Mode II crack propagation, though local curvature in the plies visible in Figure 18 which will also hinder crack advance. The scatter in Mode II values is quite low; the crack tends to stay at the same 0/90° interface during propagation.

**Figure 19:**
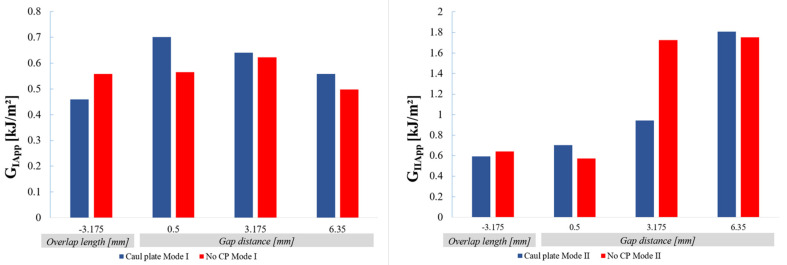
Influence of the caul plate on Mode I (**left**) and Mode II (**right**) fracture energies.

Finally, concerning the influence of the caul plate, the data are provided in Table 1 and Table 2, but Figure 19 shows plots of mean values to illustrate the effects more clearly.

Under Mode I loading the caul plate tends to increase the fracture energy slightly, perhaps due to a more constrained interlaminar microstructure resulting in less planar interfaces.

Under Mode II loading the difference is small for small defects but for larger gaps (3.175 mm) the crack resistance with a caul plate is lower than without. For the largest 6.25 mm gaps the results are similar with and without the caul plate. This suggests there may be a critical ratio between the dimensions of the zone affected by the defect and the homogenizing effect of the caul plate, which controls the damage zone and energy required to propagate the crack. More work is needed to quantify this effect.

## 5. Conclusions

This study investigates the effect of AFP manufacturing gap/overlap singularities on the crack propagation behaviour in carbon fibre epoxy laminates. Such local defects may occur due to the variation of tape width or laying tapes over complex shapes while their influence under in-plane loads was characterized in two previous studies [17,18]. Here original results for out-of-plane loads are presented for the same materials and defects.

It was first necessary to develop a specific test procedure and laminate stacking sequence. Mode I and Mode II interlaminar fracture tests were performed because results from simpler tests (ILSS) were shown not to be sensitive to these defects. The latter test involves a complex stress field, with compression tending to close the defects, and should not be used to investigate the influence of AFP manufacturing singularities.

The interlaminar fracture results show clear effects of the gap defects on the crack propagation behaviour under out of plane loading. The effects measured increase with increasing defect size. The influence of these defects under both Mode I and Mode II loading conditions is always to slow down crack propagation, promoting unstable crack growth. Use of a caul plate during manufacturing influences the measured values of fracture energy but not the overall trends observed. 

In further work, it would be interesting to examine whether cyclic and dynamic loading have a similar influence on the fracture behaviour.

## Figures and Tables

**Table 1:** Summary of Mode I test results. Mean values and standard deviations from tests on 6 specimens for each configuration.

	Mode I Delamination Toughness (kJ/m^2^)	
	Embedded Defect Region	Propagation a = 80 mm
Gap 0.5 mm—Without CP	0.565 (±) 0.136	0.425 (±) 0.090
Gap 0.5 mm—With CP	0.701 (±) 0.137	0.371 (±) 0.073
Gap 3.175 mm—Without CP	0.623 (±) 0.098	0.431 (±) 0.127
Gap 3.175 mm—With CP	0.641 (±) 0.196	0.384 (±) 0.061
Gap 6.35 mm—Without CP	0.498 (±) 0.177	0.373 (±) 0.055
Gap 6.35 mm—With CP	0.558 (±) 0.117	0.353 (±) 0.068
Overlap 3.175 mm—Without CP	0.559 (±) 0.141	0.389 (±) 0.099
Overlap 3.175 mm—With CP	0.460 (±) 0.071	0.402 (±) 0.014

CP: Caul Plate.

**Table 2:** Summary of mode II test results. Mean values and standard deviations from tests on 6 specimens for each configuration.

	Mode II Delamination Toughness (kJ/m^2^)	
	Embedded Defect Region	Propagation a = 75 mm
Gap 0.5 mm—Without CP	0.576 (±) 0.052	0.447 (±) 0.079
Gap 0.5 mm—With CP	0.702 (±) 0.037	0.583 (±) 0.075
Gap 3.175 mm—Without CP	0.1727 (±) 0.130	0.725 (±) 0.074
Gap 3.175 mm—With CP	0.944 (±) 0.069	0.472 (±) 0.045
Gap 6.35 mm—Without CP	1.752 (±) 0.112	0.641 (±) 0.070
Gap 6.35 mm—With CP	1.809 (±) 0.135	0.625 (±) 0.079
Overlap 3.175 mm—Without CP	0.642 (±) 0.088	0.461 (±) 0.067
Overlap 3.175 mm—With CP	0.592 (±) 0.089	0.434 (±) 0.038

CP: Caul Plate.

## Data Availability

Raw data confidential at present, study ongoing.
